# Reflections upon human cancer immune responsiveness to T cell-based therapy

**DOI:** 10.1007/s00262-012-1274-9

**Published:** 2012-05-11

**Authors:** Ena Wang, Sara Tomei, Francesco M. Marincola

**Affiliations:** Infectious Disease and Immunogenetics Section (IDIS), Department of Transfusion Medicine, Clinical Center and Trans-NIH Center for Human Immunology (CHI), National Institutes of Health, Bldg 10, Room 1C711, 9000 Rockville Pike, Bethesda, MD 20892 USA

**Keywords:** CIMT 2011, Melanoma, Tumor immunotherapy, Immunoresponsiveness, Cancer vaccines

## Abstract

Immune-mediated rejection of human cancer is a relatively rare but well-documented phenomenon. Its rate of occurrence progressively increases from the occasional observation of spontaneous regressions to the high rate of complete remissions observed in response to effective treatments. For two decades, our group has focused its interest in understanding this phenomenon by studying humans following an inductive approach. Sticking to a sequential logic, we dissected the phenomenon by studying to the best of our capability both peripheral and tumor samples and reached the conclusion that immune-mediated cancer rejection is a facet of autoimmunity where the target tissue is the cancer itself. As we are currently defining the strategy to effectively identify the mechanisms leading in individual patients to rejection of their own tumors, we considered useful to summarize the thought process that guided us to our own interpretation of the mechanisms of immune responsiveness.

## The molecular basis of T cell-mediated immune recognition and the self–non-self preposition

The first evidence that T cells can recognize cancer was based on the description by Wolfel et al. [1] of tumor infiltrating lymphocytes (TILs) that could kill autologous tumor cells following a human leukocyte antigen (HLA)-restricted pattern and by Kawakami et al. [2] who characterized the requirements for HLA restriction. In 1991, the molecular characterization of the first tumor antigen (TA) recognized by T cells was done by Van der Bruggen et al. [3]. This discovery, followed by the identification or several other TA-specific molecular targets of T cells [4, 5], provided the opportunity for tumor-specific immunotherapeutic intervention [6]. Contemporaneously, others identified naturally occurring antibodies directed against TAs of which several were also recognized by T cells [7].

One category of TAs included melanoma differentiation antigens (MDA) whose expression is shared by melanoma cells and normal epithelial melanocytes. Another category includes proteins whose expression was associated with the progressive de-methylation of cancer cells; as these non-mutated proteins were expressed selectively by germ line cells in the testes and cancer cells, they were named cancer testing antigens (CTA).

The identification of MDAs and CTAs and their respective epitopes associated with specific HLA molecules spurred interest in using these molecules and their derivatives for TA-specific active immunization [8]. An increasing body of literature has more recently focused on TA derived from infectious agents such as human papilloma virus or immunogenic mutated proteins whose expression may or may not be shared by different patients’ tumors depending upon the frequency of a given mutation. These TAs may represent a distinct and important facet of T cell-mediated recognition of tumors. Indeed, recent work suggests that immunization against viral antigens may be particularly promising [9]. The current viewpoint represents a retrospective evaluation of what we learnt predominantly studying MDA-specific vaccinations; we believe that part of the learning may apply to a broader range of phenomena related to T cell-based immunotherapy of cancer while in other cases, more immunogenic non-self TA may stand on their own. Future work should include of course these approaches for which at the moment there is relatively less clinical experience.

## Epitope-specific anti-cancer vaccines as a model to understand immune rejection

Despite the large number of trials vaccinating patients with TAs, results have been in general disappointing [8, 10, 11], although in a minority of cases, vaccines may prolong the frequency of clinical responses and/or survival [12–14]. One of them is now the first anti-cancer vaccine licensed by the Food and Drug Administration. Independent of their clinical potential, epitope-specific anti-cancer vaccines, however, provided to immunologists the unprecedented opportunity to study in relatively controlled settings how the human immune system functions. Thus, it could be tested whether vaccine-induced circulating TA-specific T cells could reach the tumor site and recognize their target [15]; the use of tetrameric HLA complexes [16] made it possible to characterize ex vivo the functional properties of vaccine-induced T cells [17, 18].

## The paradoxical co-existence of tumor-specific T cells (in the circulation and in the tumor) and their target

Anti-cancer vaccination efforts consistently elicited expansion of TA-specific memory T cells in the peripheral circulation, but this was rarely accompanied by cancer regression [19], suggesting that tumor rejection is a complex phenomenon that goes beyond the recognition of target cells by T cells [20]. Several questions emerged: do vaccine-induced T cells reach the tumor site? Does the tumor microenvironment provide sufficient co-stimulation to activate otherwise quiescent T cells [21]? How do the evolving phenotypes of cancer cells escape potentially effective immune responses? Most intriguingly, are tumor-changing phenotypes responsible for cancer cell escape from recognition by an adequately activated immune response? Alternatively, are the immune responses elicited by vaccines insufficient to destroy cancer cells that are otherwise adequately expressing target molecules [21]?

## The quiescent status of vaccine-induced T cell

Lee et al. [22] documented a status of anergy of spontaneously occurring MDA-specific circulating T cells. Further work demonstrated that, beyond stage II, patients with breast and colon cancer or melanoma suffer a profound depression of innate immune responses. This immune suppression is exemplified by reduced production of IFN-γ in response to TA-specific stimulation ex vivo [22] and reduced inducible levels of signal transducer and activator of transcription (STAT)-1 phosphorylation in circulating immune cells following ex vivo stimulation with type I interferon (IFN) [23, 24]. Interestingly, it was observed that although patients with cancer experience a significantly reduced responsiveness to IFN-α stimulation compared with healthy individuals, such depression occurs within a big range of values with some patients demonstrating normal response to stimulation also at a later stage of disease. This suggests that some aspects of the host’s or the individual tumor biology may differ dramatically among different cancer-bearing subjects. Similar findings were later reported by others, who observed in patients with cancer the same depression in STAT-1 phosphorylation following stimulation with IL-2 [25]. It also became apparent that spontaneously occurring anti-cancer immune responses in patients with metastatic melanoma displayed a memory phenotype [26, 27] providing evidence for in vivo priming of effector T cells by the cancer-bearing status [26].

While the work of Lee and other groups focused on spontaneously occurring TA-specific T cells, others analyzed the type and function of vaccine-elicited T cells documenting that naturally occurring TA-specific immune responses could be consistently enhanced by immunization [28, 29]. Contrary to naturally occurring TA-specific T cells incapable of expressing IFN-γ in response to cognate stimulation [22], vaccine-induced T cells were able to produce IFN-γ ex vivo [17]. However, they lacked cytotoxic function that could be recovered by in vitro activation with TA-specific stimulation in the presence of recombinant interleukin (IL)-2. We defined these incompletely anergic vaccine-induced T cells as “quiescent” [18]. This quiescent phenotype was reminiscent of the contraction phase of effector immune responses described by Kaech [30] according to the linear model of T cell activation. According to this model, following a time-delimited stimulation, T cells expand and become activated during the first week, acquiring also cytotoxic properties. In the following 2–3 weeks, CD8 T cells contract in number and, while maintaining responsiveness to antigen-specific stimulation, loose cytotoxic function. This model well approximates the kinetics of TA-specific immunization, which is provided at intervals of 1–3 weeks, and T cell immune responses are generally tested weeks after immunization. This observation raised the question: assuming that vaccine-induced T cells reach the tumor site, could the tumor in steady state conditions or during immune therapy provide a *milieu* conducive to their reactivation comparable to that simulated in vitro by re-stimulation with TA in the presence of IL-2 [18]? While most remained interested in the afferent aspects of vaccination and continued to develop increasingly more sophisticated strategies aimed at inducing qualitatively and quantitatively better immune responses, we turned our interest toward the efferent loop to understand the requirements for their activation in the target organ. This was based on the assumption that circulating vaccine-induced T cells were fully functional according to the physiology and the dynamics of T cell activation in response to a time-limited stimulus such as a vaccine [21]. It should be clarified that the afferent aspect of immunization include mode of action studies and the pharmacodynamics that are integral part of a rational development of immune therapies aiming at inducing specific immune responses while the efferent aspect includes the study of those variables that determine the effects of vaccine-induced immune responses after their induction. It should also be emphasized that the study of the afferent loop of tumor vaccines remains important for drug developers aiming to understand the mechanism of action of their products; many basic questions remain unsolved (which antigen format, which adjuvant, which route of administration, which dosing schedule, etc.). Thus, although drug developers made in the past the mistake of concentrating mostly on the afferent loop neglecting the efferent one, we should not make the reverse mistake now but rather combine the two approaches into an integrated and systematic analysis of all potential variables that could decode the algorithm governing immune responsiveness.

## The limited role of tumor immune escape as predictor of immune responsiveness

**Fig. 1:**
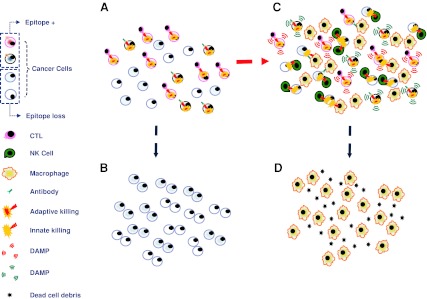
Extremes in the interpretation of the mechanisms leading to tumor rejection and their impact on the relevance of the heterogeneity of tumor cells in determining their escape from immune recognition. **a** In the simplest scenario, adaptive immune responses, whether mediated by cytotoxic T cells (*CTLs*) or antibody-mediated cytotoxicity, play a prominent role in eliminating cancer cells expressing the appropriate epitope; in a cancer population with heterogeneous expression of target epitope due to loss of antigen or antigen processing and presentation defects, only the epitope expressing cancer cells would be eliminated leaving the other ones alive (**b**). **c** An alternative scenario suggests that the effector/target cell complex may deliver pro-inflammatory signals due to the release of cytokines or damage-associated molecular patterns (*DAMPs*; in the figure, in *red* are those associated with T cell effector function, in *blue* those associated with antibody-mediated cytotoxicity). These, in turn, attract and activate innate immune effectors such as natural killer (*NK*) cells or macrophages which can exert cytotoxic functions on cancer cells independent of epitope expression. This, in turn, may lead to a broader clearance of cancer cells leaving only macrophages in charge of tissue repair (**d**). Which of these scenarios more closely represent human reality remains to be determined

Evolution under selection implies that forceful negative pressure is imposed upon malleable phenotypes. To explain the co-existence of a cognate immune response against cancer with its concurrent growth, immune selection is often invoked [31, 32]. This concept revolves around the demonstration in experimental and clinical models that immune suppression is associated with higher incidence of spontaneous cancers [33, 34]. Recently, this concept is enjoying broader clinical recognition as the relevance of immune infiltrates on the natural history of cancer is becoming increasingly appreciated [35, 36]. To explain why tumors grow in the presence of an active immune response, the work of several groups including ours has described and characterized several potential mechanisms by which tumors could escape immune recognition [20, 32, 37–39]. However, these observations refer to conditions when tumors do not undergo immunotherapy and a balance is stricken between the host’s immune response and cancer growth. Therefore, it is likely that immune escape, also referred to as immune editing [40], may play a significant role at the onset and throughout the natural history of cancer. However, to establish causation in the determinism of responsiveness to immunotherapy, level of TA and TA presentation should be assessed before treatment and a predictive relationship should be demonstrated on the likelihood to respond to the respective treatment. In fact, the balance stricken between the host’s immune response and its cancer in natural conditions is shaken by powerful immune responses observable during an acute inflammatory process [41]. Thus, the described mechanism that could explain reduced recognition of tumor cells in natural conditions may not apply to the lack of tumor responsiveness to effective immunotherapy when the balanced is switched suddenly in favor of the host, and cancer cells had no time to adapt to the novel condition. As later described, direct human observations of tumors performed before treatment did not demonstrate that the level of TA expression predicts response to therapy. Those studies suggested that the lack of response to immunotherapy is due to limited activation of immune responses within the target organ rather than the loss of antigenic properties by cancer cells. Loss of TA expression appears only after treatment in recurring lesions following partially successful therapy that did not completely eradicate all cancer cells [20] (Fig. 1).

## On the necessity to study the tumor microenvironment and the serial biopsy strategy

Direct observation of the tumor microenvironment may obviate for the need of hypotheses [42, 43]. As TSD is tissue-specific, it is best to study the target organ: the cancer itself. There is a dissonance between the current emphasis on the characterization of circulating immune cells and the indirect role that they play in the cancer microenvironment. The approach lacks appreciation for the heterogeneity of individual tumors and their dynamic instability [44]. Thus, to understand human cancer immune responsiveness, tumors should be studied in steady state conditions to document patient-specific idiosyncrasies and during therapy to discover phenomena relevant to TSD. It should be kept in mind that immune manipulation of the host may not only effect the function of immune cells but, directly or indirectly, the function also of cancer cells or other normal cells within the tumor microenvironment. It is only during those dynamic moments that the mechanisms relevant to rejection will emerge; only at those moments, modulation of HLA expression [45–48] or changes in cytokines-dependent modulation of the immune microenvironment become apparent as well as exemplified by the dual role played by IL-10 that will be discussed later [49]. Thus, we conclude that tumor-related TSD could be effectively studied by catching the switch from a chronic to an acute inflammatory process by serially studying individual tumors before, during, and after treatment [50].

It soon became apparent, however, the classical approaches to the study of tumors were not suitable. Most studies utilized excisional biopsies, which provide optimal quantity of tissue and allow its morphological assessment. However, because the tumor is removed, serial sampling of the same lesion for dynamic studies cannot be done. Moreover, no direct correlation can be performed between the findings obtained studying the excised material and the response to immunotherapy of concomitant metastases left in the patient. The assumption that removed tumors are representative of those left in the patient as targets of therapy was soon proved inaccurate; the study of synchronous metastases demonstrated significant variability among most markers evaluated [51]. We, therefore, applied in subsequent studies minimally invasive biopsies such as fine needle aspirate (FNA) biopsies that allowed serial sampling of the same lesions [50].

## Localization of vaccine-induced T cells at tumor site may be necessary but it is not sufficient for tumor regression

Localization within tumors of indium^111^-labeled adoptively transferred TILs is necessary for clinical response [52]; however, TIL localization is not sufficient, and several lesions demonstrating uptake do not respond to therapy.

Since TIL localization in the target organ is necessary for tumor rejection, we tested whether vaccine-induced T cells reached the tumor site. Kammula et al. [15] applied serial FNAs to melanoma metastases, comparing the expression of interferon (IFN)-γ before and during vaccination. This study was performed on lesions whose cancer cells did express sufficient amount of the relevant HLA allele to exclude escape from T cell recognition; expression of the MDA target of the vaccination was monitored in parallel. Furthermore, localization of vaccine-specific T cells was monitored with tetrameric HLA/peptide complexes. This study clearly documented a significant enhancement in IFN-γ expression in tumors following vaccination, and the level of expression of this cytokine correlated with the level of expression of the relevant MDA. Thus, vaccine-induced T cell do reach the tumor site, interact with tumor cells by producing IFN-γ and, therefore, are not anergic. However, this functionally competent localization was not sufficient for tumor rejection as all lesions continued to grow. This finding mirrors the quiescent phenotype of vaccine-induced T cells observed in the circulation that could produce IFN-γ when encountering the relevant TA but cannot exert broader effector functions.

## The surprising role of IL-10

Several studies suggest that tumors produce immune regulatory molecules that could suppress the function of T cells. Applying FNA biopsies, we assessed in pre-treatment samples the expression of cytokines with potential regulatory effects [53]. Surprisingly, the expression levels of none of them predicted unresponsiveness; on the contrary, higher levels of IL-10 significantly predicted response. Further analyses demonstrated that the IL-10 was not only expressed at the RNA but also at the protein level by cancer cells. Retrospectively, this observation was not surprising as several other studies had observed that, contrary to the expectations, IL-10 overexpression was a predictor of immune responsiveness. This cytokine probably favors tumor growth in steady state conditions by inhibiting the maturation of antigen presenting cells, but at the same time, sustaining their ability for antigen uptake and simultaneously hampering their migration to draining lymph nodes prepares antigen presenting cells to serve as powerful T cell stimulators loaded with TA when the microenvironmental conditions are altered during immune stimulation that could affect a switch of their phenotype by the presence of immune stimulatory cytokines such as IFN-γ and TNF-α that could induce their maturation in situ [49].

## Levels of HLA/TA expression by cancer cells does not predict immune response

It is logical that the success of TA-specific vaccination is predicated upon the level of expression of the target TA by cancer cells. We, therefore, measured by FNA in pre- and post-treatment metastases the expression of TA relevant and irrelevant to the vaccine administered [54]. The analysis included only melanoma metastases whose cancer cells expressed the associated HLA molecule necessary for T cell recognition. The level of expression of the target TA was not predictive of responsiveness. However, we observed that lesions biopsied soon after treatment as they were undergoing a biological response that subsequently led to their complete disappearance dramatically reduced the expression of the TA target of the vaccine. This observation suggests that when immunotherapy works, the first targets of therapy are the cancer cells expressing the relevant TA. The loss of TA expression was not associated with complete disappearance of cancer cells at that time point as other TA expressed specifically by cancer cells such as CTAs were still present, and cancer cells could still be observed in the cytospins. Thus, loss of cancer cells expressing TA targeted by the vaccine preceded the elimination of the complete neoplastic population. Since the metastases subsequently underwent complete disappearance, it became clear that the effect of vaccination was to initiate a self-perpetuating process that continued beyond the initial interaction between vaccine-induced T cells and their targets [20, 55]. The same conclusion was subsequently independently reached by others in the context of CTA-specific anti-cancer vaccination [56].

## The advent of whole-genome, hypothesis-generating studies

The dynamic approach to the study of immune responsiveness had, by the end of the 1990s, crushed several myths. Hundreds of modulatory mechanisms that could hamper immune responsiveness could be postulated but such number was too high to be realistically tackled by future studies with a minimalistic approach. Around that time, whole-genome transcriptional assays enter the scene of clinical investigation [57]. The hypothesis-generating strategy well suited our lack of a dominant hypothesis and proved invaluable for the application of an inductive approach aimed at decoding the multifactorial algorithm governing tumor immune responsiveness [41, 43, 50, 55, 58–60]. In particular, we became interested in characterizing two phenomena: first, document the mechanism of tumor rejection by sampling lesions during and after treatment and comparing those that eventually responded to those that did not. Second, dissect the reasons why some but not all tumors respond to identical treatments.

## The pre-determinism of immune responsiveness

The first attempt to address at the whole genome level the determinism leading to tumor rejection was an analysis of melanoma metastases biopsied before and after treatment with TA-specific vaccination and concomitant systemic administration of IL-2 [45]. Analysis of pre-treatment biopsies identified a set of genes differentially expressed by lesions that subsequently responded to treatment. Although those early array platforms contained few immunologically relevant genes, the transcripts that differentiated responding from non-responding metastases had predominantly immune function. This observation leads to the conclusion that some metastases were pre-determined to respond to immunotherapy by a pre-existing inflammatory process that although not sufficient to induce spontaneous tumor regression was conducive to immune stimulation. A decade later, we could validate in a small prospective mechanistic study these findings [47]: pre-treatment biopsies in patients with metastatic melanoma who subsequently responded to systemic IL-2 administration displayed a pre-existing pro-inflammatory status. Simultaneously, others identified similar signatures to be predictive of responsiveness in patients with melanoma vaccinated with four TAs plus IL-12 [61], dendritic cells loaded with multiple TAs [62], or melanoma and lung cancer patients receiving a MAGEA3 peptide vaccine [63].

## Similarity between cancer rejection and other aspects of immune-mediated tissue-specific destruction (TSD)

In a separate study, we evaluated the mechanism of action of systemic IL-2 administration by sampling melanoma metastases before and during treatment. This study demonstrated that the mechanism of action of IL-2 is an indirect activation of macrophages mediated by a cytokine storm released by circulating IL-2 receptor-bearing cells [64]. The effects at the tumor site were the degrees of magnitude less than at the systemic level. Moreover, the intra-tumor effects were delayed, and the magnitude was dose dependent. This could explain why immune responsiveness has been associated with number of doses received by patients [65]. Because one of the lesions from the six patients studied underwent clinical response, we had the first glimpse at the transcriptional machinery specific for tumor rejection [64]. Surprisingly, several of the transcripts expressed specifically by the responding lesion had been simultaneously described by another group as markers of acute kidney allograft rejection [66]. This was the first suggestion that tumor regression, allograft rejection, and probably other immune destructive processes were facets of a common phenomenon that shared a convergent final pathway.

## From the delayed allergy reaction to the immunologic constant of rejection (ICR)

In 1969, Jonas Salk [67] hypothesized that allograft rejection, tumor rejection, and various autoimmune phenomena represented facets of a similar immune-mediated phenomenon that he called the “delayed allergy reaction”. His intuition was validated decades later by work from those who studied with high throughput technology tissue undergoing TSD. This unbiased approach applied to acute allograft rejection, tissues affected by graft versus host disease or flare of autoimmunity, acutely infected organs undergoing clearance of pathogen and tumor rejection during immunotherapy identified a convergent pathway and a group of genes that were always required for TSD to occur. We named this signature “the immunologic constant of rejection (ICR)” [55]. The ICR includes at least 4 functional components: the activation of (1) the IFN-γ/STAT1/IRF-1 pathway [41], (2) immune effector mechanisms, (3) CXCR3, and (4) CCR5 ligand chemokines [68].

## How defining the ICR guided the understanding of immune responsiveness

The ICR axiom describes how TSD occurs independent of its cause; in other words, the ICR dictates “how” but not “why” rejection occurs. However, the definition of a necessary pathway clarified the premises for the identification of its causes. It became clear that the lack of rejection of tumors during immunotherapy is due to insufficient stimulation of immune responses at the tumor site rather than to the escape of tumors from a fully activated immune response. Analysis of patients with melanoma experiencing a mixed responses [48] and comparison of tumors responding and non-responding to the same treatment [45, 47] demonstrated that while responding tumors displayed a strongly activated acute inflammatory status, non-responding ones were completely immunologically silent as if the treatment had not reached the target tissue. Moreover, the definition of the ICR leads to the conclusion that genes and pathways activated during TDS are qualitatively similar to those associated with a good prognostic connotation in various cancers [35, 36, 69] and to those predicting immune responsiveness to distinct type of immunotherapy [45, 47, 61, 63, 70]. Thus, a general theory of rejection could be constructed, whereby a progression occurs in the host versus cancer relationship, starting with immune surveillance that slows but does not abrogate tumor growth to a pre-determinism to respond to therapy to a full blown activation of the immune response leading to cancer rejection during treatment. Similar signatures are observed throughout this continuum but the intensity and breath of their activation increases progressively till TDS is reached.

## A genetic inference on human cancer immune responsiveness; the role of the genetic background of the host, neoplastic instability, and external factors

Perhaps, the most important contribution offered by the ICR concept in deconvoluting the determinism of tumor rejection is the provision of a road map defined by evidence-based hypotheses. Identification of a specific group of genes, whose activation is necessary for TSD, leads to the analysis of the role of IRF5 polymorphism in melanoma immune responsiveness. This study was based on the premise that, if the ICR applies to autoimmunity and if cancer rejection represents an aspect of autoimmunity, genes relevant to the development of autoimmunity ought to influence the immune biology of cancer. IRF5 plays a significant role in the modulation of several autoimmune diseases including systemic lupus erythematosus (SLE). We recently observed that the same polymorphisms protective against the development of SLE predict non-response to adoptive transfer of TILs (unpublished own data). Similarly, appreciation for the central role played by the CXCR3/CCR5 cluster in mediating TSD leads us to the investigation of its expression by TILs. Integration of germ line, transcriptional, and protein expression data brought to the formulation of a protein prediction model strongly predictive of treatment outcome (unpublished own data). It is important to note that in this study, the expression of individual receptors was not highly predictive of immune responsiveness but the combined over- or underexpression was strongly pointing to the need to look at the biomarkers of immune responsiveness not as individual entities but in accordance with their combined function. Importantly, this second-generation, evidence-based, and hypothesis-driven studies enlightened an underappreciated fault of current bioinformatics approaches applied to the identification of biomarkers of immune responsiveness. This study demonstrated that individual, univariate approaches bear, when examined individually, very little predictive value but the combination of factors integrated according to a logical stream may progressively break the code governing the algorithm of immune responsiveness.

## The genetics of the host, the tumor, and the environment

The relative weight played by the host’s genetic background, the somatic alterations acquired during the neoplastic process, and the influence of environmental factors in determining immune responsiveness is unclear. Observations performed during the last two decades strongly suggest that it is a combination of the three that determine immune responsiveness [60]. It is likely that inherited genetic factors may affect the biology of the host or their cancer cells to determine the likelihood of immune responsiveness; to this first checkpoint, the genetics of cancer cells may add its own predisposition to being susceptible to immune attack. Finally, various undetermined variables encompassing the effectiveness of treatment, general status of health and other “hidden” external factors may contribute to the final outcome. Only when all checkpoints are permissive, tumor rejection is observed. Moreover, the genetic weight that the host’s background plays on tumor biology is often underestimated. Tumors from individual patients are close to each other biologically when compared to those from other patients; this may not only be due to their clonal derivation as often assumed [71, 72], but it could also depend upon the innumerable polymorphisms inherited by cancer cells from their host. Thus, the genetic background of the host may affect immune responsiveness, not only by affecting the function of normal immune cells but also by directly affecting the biology of cancers cells. This multistep inference on the mechanism of immune responsiveness may also explain why it is generally easier to predict accurately lack of responsiveness than responsiveness; it is necessary to have a gun to go duck hunting; if no gun is available no duck will be caught; but having a gun is not sufficient as ability to shoot, good visibility, presence of ducks, and many other limiting factors may determine the success of the hunt.

## The future challenges and opportunities

In the same manuscript where the delayed allergy reaction was described, Jonas Salk [67] also stated: “The answers pre-exist and are the questions that need to be identified”. We totally agree. The algorithm governing tumor response to immunotherapy is lying in front of us. The recipe to its identification is simple: first, study the tumor together with the peripheral circulation; second, study the genetic background of the host together with the genetics of the tumor; third, apply integrative bioinformatics approaches searching for complex relationships rather than univariate class comparisons. We are confident that this recipe will soon lead to the understanding of tumor immune responsiveness with a caveat: we need to sensitize those who design clinical trials to the need to learn from clinical investigations rather than limiting the testing to the clinical endpoint [73].

## Abbreviations

CTA: Cancer testing antigen
FNA: Fine needle aspiration biopsy
HLA: Human leukocyte antigen
ICR: Immunologic constant of rejection
IEFs: Immune effector genes
IFN: Interferon
IL: Interleukin
IRF: Interferon regulatory factor
ISGs: Interferon-stimulated genes
MDA: Melanoma differentiation antigens
SLE: Systemic lupus erythematosus
STAT-1: Signal transducer and activator of transcription
TIL: Tumor infiltrating lymphocyte
TA: Tumor antigen
TSD: Immune-mediated tissue-specific destruction
